# Reply to the Letter on: “Electrophysiological and Biochemical Evaluations of Neuropathy Risk in Oral Levodopa versus Levodopa/Carbidopa Intestinal Gel Treatment”

**DOI:** 10.1055/s-0046-1825517

**Published:** 2026-09-15

**Authors:** Miray Erdem, Bugra Selluncak, Mehmet Balal, Halit Fidanci, Meltem Demirkiran

**Affiliations:** 1 Health Sciences University, Adana City Training and Research Hospital, Department of Neurology, Adana, Türkiye.; 2 Cukurova University, Faculty of Medicine, Department of Neurology, Adana, Türkiye.; 3 Health Sciences University, Adana City Training and Research Hospital, Department of Neurology, Division of Neurophysiology, Adana, Türkiye.

Dear Editor,

We would like to thank Mahida and Jagtap for their thoughtful and constructive comments regarding our study on electrophysiological and biochemical markers of neuropathy risk in Parkinson's disease patients treated with oral levodopa and levodopa/carbidopa intestinal gel. 1 We appreciate the authors' careful interpretation of our findings and their emphasis on several important methodological considerations, including levodopa exposure intensity, electrophysiological heterogeneity, and the cross-sectional nature of the biochemical correlations.

As correctly highlighted, differences in levodopa equivalent daily dose may contribute to metabolic alterations such as homocysteine elevation, which, in turn, could influence peripheral nerve function. 2 3 4 5 We agree that dose-normalized analyses and longitudinal observations would further refine the interpretation of these associations.

Regarding the neurophysiological findings, we acknowledge that subclinical polyneuropathy in Parkinson's disease typically follows a length-dependent and symmetrical pattern. In our study, the electrophysiological protocol was designed to detect early or subtle abnormalities across multiple nerves; however, we agree that composite neuropathy indices and standardized diagnostic criteria may further strengthen the characterization of diffuse neuropathy in future studies. 2 3 4 5

Importantly, motivated by the questions raised in this field, we have initiated an ongoing prospective study including newly-diagnosed Parkinson's disease patients who have not yet received any dopaminergic treatment. In this cohort, comprehensive electrophysiological assessments are performed at baseline and repeated after 1 year of follow-up. In addition to expanding the electrophysiological evaluation, the study also incorporates assessments targeting small-fiber neuropathy, enabling a broader characterization of peripheral-nerve involvement over time.

We hope that the results of this ongoing work will contribute to clarifying the temporal relationship involving dopaminergic treatment exposure, metabolic alterations, and peripheral neuropathy development in Parkinson's disease. We look forward to sharing these findings in the near future.
